# Neuroprotective effects and possible mechanisms of berberine in animal models of Alzheimer’s disease: a systematic review and meta-analysis

**DOI:** 10.3389/fphar.2023.1287750

**Published:** 2024-01-08

**Authors:** Lijuan Dan, Yanwei Hao, Jiaxin Li, Tianyuan Wang, Weiwei Zhao, Hui Wang, Liyan Qiao, Peijun Xie

**Affiliations:** ^1^ School of Clinical Medicine, Hospital of Chengdu University of Traditional Chinese Medicine, Chengdu, China; ^2^ Department of Geriatrics, Hospital of Chengdu University of Traditional Chinese Medicine, Chengdu, China; ^3^ Traditional Chinese medicine department, 363 Hospital of Chengdu, Chengdu, China; ^4^ Department of Geriatrics, Xi’an Hospital of Traditional Chinese Medicine, Xi’an, China

**Keywords:** Alzheimer’s disease, Berberine, meta-analysis, animal model, systematic review

## Abstract

**Background:** Recently, multiple preclinical studies have reported the beneficial effect of berberine in the treatment of Alzheimer’s disease (AD). Nevertheless, the neuroprotective effects and possible mechanisms of berberine against AD are not universally recognized. This study aimed to conduct a systematic review and meta-analysis by integrating relevant animal studies to assess the neuroprotective effects and potential mechanisms of berberine on AD.

**Methods:** We systematically searched PubMed, Embase, Scopus and Web of Science databases that reported the effects of berberine on AD models up to 1 February 2023. The escape latency, times of crossing platform, time spent in the target quadrant and pro-oligomerized amyloid beta 42 (Aβ_1-42_) were included as primary outcomes. The secondary outcomes were the Tau-ps 204, Tau-ps 404, β-site of APP cleaving enzyme (BACE1), amyloid precursor protein (APP), acetylcholine esterase (AChE), tumor necrosis factor ⍺ (TNF-α), interleukin 1β (IL-1β), IL-6, nitric oxide (NO), glial fibrillary acidic protein (GFAP), malonaldehyde (MDA), glutathione S-transferase (GST), glutathione (GSH), glutathione peroxidase (GPx), Beclin-1 and neuronal apoptosis cells. This meta-analysis was conducted using RevMan 5.4 and STATA 15.1. The SYRCLE’s risk of bias tool was used to assess the methodological quality.

**Results:** Twenty-two studies and 453 animals were included in the analysis. The overall results showed that berberine significantly shortened the escape latency (*p* < 0.00001), increased times of crossing platform (*p* < 0.00001) and time spent in the target quadrant (*p* < 0.00001), decreased Aβ_1-42_ deposition (*p* < 0.00001), Tau-ps 202 (*p* < 0.00001) and Tau-ps 404 (*p* = 0.002), and improved BACE1, APP, AChE, Beclin-1, neuronal apoptosis cells, oxidative stress and inflammation levels.

**Conclusion:** Berberine may be a promising drug for the treatment of AD based on preclinical evidence (especially when the dose was 5–260 mg/kg). The potential mechanisms for these protective effects may be closely related to anti-neuroinflammation, anti-oxidative stress, modulation of autophagy, inhibition of neuronal apoptosis and protection of cholinergic system. However, these results may be limited by the quality of existing research. Larger and methodologically more rigorous preclinical research are needed to provide more convincing evidence.

## 1 Introduction

Alzheimer’s disease (AD) is a progressive neurodegenerative disorder with memory deficits and cognitive impairment as its main clinical manifestations, accounting for about 60%–80% of all dementia cases (Rostagno, 2022). The pathobiology of AD is complex, among which senile plaques formed by deposition of large quantities of β-amyloid (Aβ) outside of brain neuronal cells and intracellular neuroprogenitor fibril tangles (NFTs) induced by hyperphosphorylation of tau proteins are the main neuropathological criteria for the diagnosis of AD (Edison et al., 2008; Roe et al., 2013), and they ultimately lead to neuronal degeneration, cell death and brain shrinkage (Chen and Mobley, 2019). The majority of AD cases are concentrated in people over the age of 65 and result in death approximately 7–10 years after the onset of symptoms (de Souza et al., 2018). Given the expected trend of population ageing and growth, the number of AD cases is expected to increase significantly (Collaborators, 2022). The World Alzheimer’s Disease Report 2021 states that “dementia has become the seventh leading cause of death globally, with approximately 55 million people living with dementia worldwide, and the number of people living with this disease is expected to reach approximately 80 million by 2030” (Iulita et al., 2023). This could put serious strain on the healthcare system and place a heavy socio-economic burden on society (Cummings et al., 2014). To the best of our knowledge, only five drugs have been approved by FDA for the treatment of AD from 1993-2003, including four cholinesterase inhibitors (tacrine, donepezil, rivastigmine, and galantamine) and an N-methyl-D-aspartate receptor antagonist (memantine) (Tanzi, 2021). However, the clinical effectiveness of these drugs is not satisfactory (Editorial, 2016). In recent years, more than 20 compounds have completed large phase 3 randomized, double-blind controlled trials in patients with different stages of AD, and none have shown any efficacy in slowing cognitive decline or improving overall function (Long and Holtzman, 2019). The FDA has recently approved a monoclonal anti-Aβ oligomers antibody (aducanumab). However, the rationale for approval and the extent of the clinical benefit of the antibody is under intense debate (Karran and De Strooper, 2022). In this context, there is an urgent need for alternative and complementary therapies for AD.

Traditional Chinese medicine (TCM) has been used in the clinical prevention and treatment of dementia for thousands of years, and has a wide range of pharmacological effects and low toxicity (Baker and Alvi, 2004). The earliest record of *Coptis chinensis* (also named *Huang Lian* in Chinese) as an anti-AD medicine can be traced back to 200 A.D. in *Shennong’s Herbal Classic*, which mentions that it is bitter in taste, cold in nature, enters the heart, liver, stomach and large intestine meridians, has the effect of clearing heat, drying dampness, purging fire and removing toxins, and can improve memory if eaten regularly. As the main active ingredient of *C. chinensis*, berberine (C_20_H_18_NO_4_^+^, Supplementary Figure S1) is widely used in a variety of diseases in clinical practice (Wang et al., 2017). It is reported that berberine has a wide range of pharmacological activities, including anti-inflammatory (Yarla et al., 2016), anti-oxidant (Feng et al., 2019), anti-cancer (Jabbarzadeh Kaboli et al., 2014), anti-diabetic (Ni et al., 2015), anti-hyperlipidemic (Pirillo and Catapano, 2015), and spatial memory enhancement effects (Wang et al., 2019). A large body of evidence suggests that berberine has neuroprotective effects against various central nervous system disorders such as psychotic depression (Fan et al., 2019), anxiety, cerebral ischemia (Zhao et al., 2021) and AD (Yuan et al., 2019).

The systematic review and meta-analysis for preclinical studies provide evidence-based support for the development of new drugs. A previous systematic review of 15 preclinical studies showed that berberine could be a promising multipotent agent to combat AD (Yuan et al., 2019). However, this study does not synthesize multiple independent, synthesizable outcome indicators for quantitative analysis. In addition, a large number of preclinical studies of berberine supplementation for AD have been widely reported in recent years. As the understanding of the pathogenesis of AD grows, it also provides new avenues for the development of new drugs (Hashimoto, 2018). Therefore, we integrated relevant animal studies for systematic review and meta-analysis to further comprehensively summarize the neuroprotective effects and potential mechanisms of berberine in AD.

## 2 Methods

We performed this study based on preferred reporting items for systematic review and meta-analysis statements (Page et al., 2021).

### 2.1 Search strategy

We retrieved four databases (including PubMed, Scopus, Embase, and Web of Science) for eligible studies that reported the use of berberine on AD up to 1 February 2023. Search terms were developed utilizing both participants of interest and interventions. To illustrate this, the search strategy for PubMed is shown in Supplementary Table S1.

### 2.2 Eligibility criteria

Inclusion criteria: 1) AD as the experimental model; 2) AD was established in various ways; 3) the treatment group only received any dose of berberine; 4) the control group only received the liquid without treatment effect or no treatment; 5) If the original study contained different dose-gradient interventions, only the results of the highest dose group were included; 6) the primary outcomes were escape latency, times of crossing platform, time spent in the target quadrant and Aβ_1-42_.

Exclusion criteria: 1) non-in vivo studies; 2) non-AD models; 3) non-rodent animal models; 4) groups without berberine treatment or no control group; 5) abstract, letter, comment; 6) duplicate publication.

### 2.3 Data extraction

Two investigators (Tianyuan Wang and Weiwei Zhao) independently extracted data from eligible articles. Prior to extracting the data, we developed a unified data extraction table that recorded the following data: 1) basic details: the article title, the name of the first author, the contact information of the corresponding author(s), and the publication year; 2) details of experimental animals; 3) methods of establishing models; 4) intervention measures of treatment group; and 5) experimental results. When the results were only presented graphically, the corresponding authors of the article were contacted by email for relevant information. If failing to receive the response, we applied the web plot digitizer soft to measure graph data.

### 2.4 Risk-of-bias assessment

The quality assessment of individual studies was carried out by two authors (Tianyuan Wang and Weiwei Zhao) independently using the systematic review center for laboratory animal experimentation (SYRCLE’s) RoB tool (Hooijmans et al., 2014). The contents of the evaluation covered ten domains of bias, relating to selection, detection, performance, attrition, reporting biases and other biases. Any discrepancies were resolved via discussion with the corresponding authors.

### 2.5 Statistical analysis

Meta-analysis was performed using Revman version 5.4.1 and STATA version 15.1, with a standardized mean difference (SMD) for continuous variable measures and a 95% confidence interval (CI) for the effect sizes of the indicators. The *I*-square (*I* ^2^) statistic was used to analyze heterogeneity. To identify sources of heterogeneity in the included studies, we performed subgroup analysis of escape latency, times of crossing platform, time spent in the target quadrant and Aβ_1-42_ based on the year of publication, animal species, drug dosage, modeling method and duration of treatment. In addition, sensitivity analysis was performed by removing each study individually from the meta-analysis results, and Egger’s test was adopted to assess potential publication bias for escape latency, times of crossing platform, time spent in the target quadrant and Aβ_1-42_. The Trim-and-fill method was performed in the presence of publication bias. To better elucidate the effect of dose and duration of administration on the results, time-dose effect relationship plots were created for escape latency, times of crossing platform, time spent in the target quadrant and Aβ_1-42_. When the drug involved different doses of administration, all dose groups with *p* < 0.05 were included.

## 3 Results

### 3.1 Study selection

**FIGURE 1:**
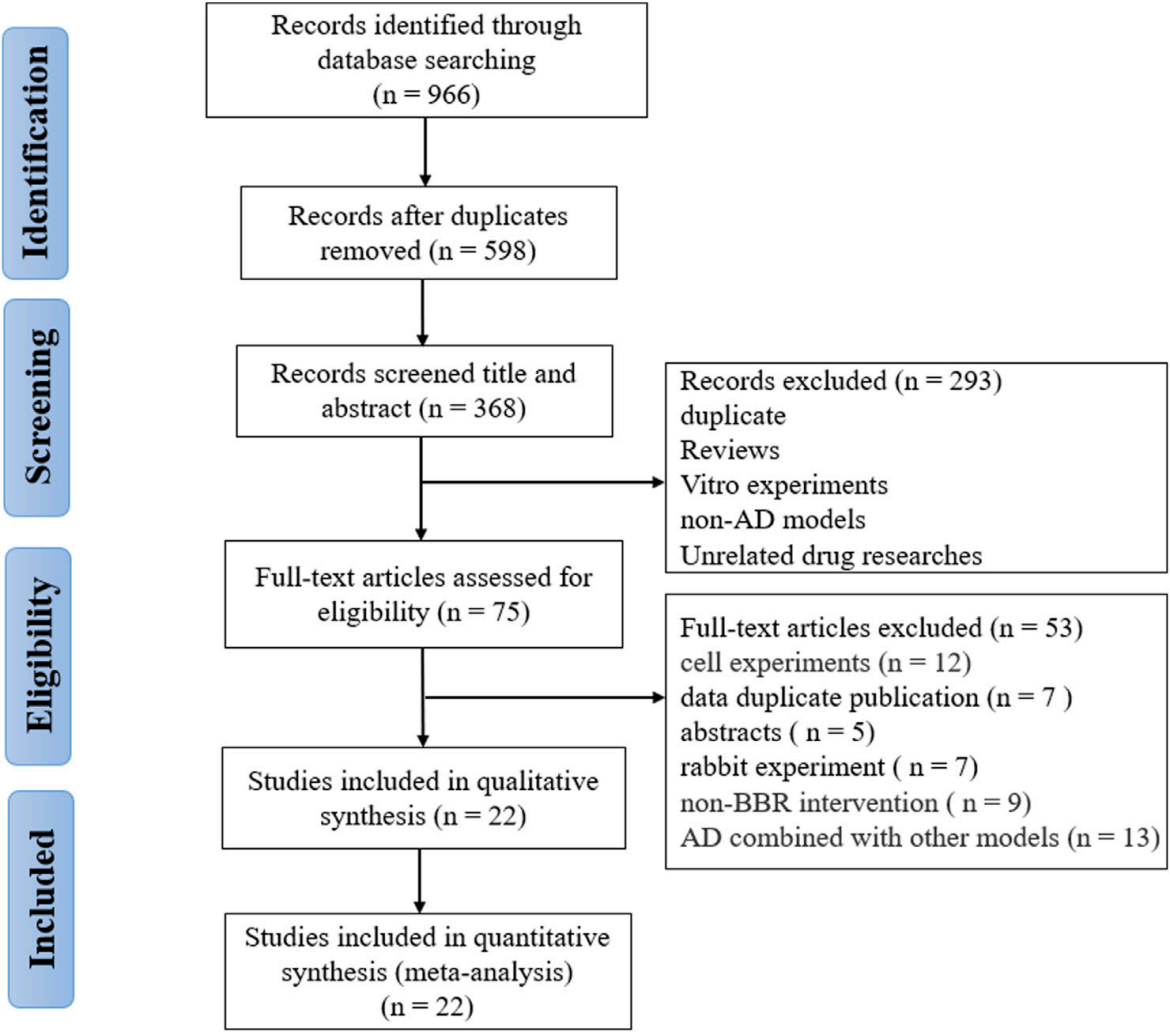
Flow diagram of the selection process.

A total of 966 studies (111 from Pubmed, 400 from Embase, 237 from Wed of Science, and 218 from Scopus) were collected through the pre-set search strategy. After removing duplications, 598 records were retained. Two reviewers (W.T.Y. and Z.W.W.) independently screened the titles and abstracts, and 293 articles were removed for the following reasons: reviews; cell experiments; unrelated models; unrelated interventions. Subsequently, we thoroughly read the full text of the remaining 75 articles and excluded 53 including non-rodent animal experiments, non-AD models, no cognitive outcomes, AD combined with other models, only abstract and data duplication. Finally, 22 eligible researches (Zhu and Qian, 2006; Durairajan et al., 2012; Lee et al., 2012; Mangrulkar et al., 2013; Haghani et al., 2015; de Oliveira et al., 2016; Mangrulkar et al., 2016; He et al., 2017; Huang et al., 2017; Cai et al., 2018; Hussien et al., 2018; Abdel-Latif et al., 2019; Cai et al., 2019; Chen et al., 2020; Lin et al., 2020; Liang et al., 2021; Raju et al., 2021; Saleh et al., 2021; Wang et al., 2021; Wu et al., 2021; Ye et al., 2021; Yang and Wang, 2022) were selected for analysis (Figure 1).

### 3.2 Study characteristics

All studies were published in ranging from 2006 to 2022, with 11 studies (50%) (Abdel-Latif et al., 2019; Cai et al., 2019; Chen et al., 2020; Lin et al., 2020; Liang et al., 2021; Raju et al., 2021; Saleh et al., 2021; Wang et al., 2021; Wu et al., 2021; Ye et al., 2021; Yang and Wang, 2022) published in the last 5 years. Detailed information on berberine in each study was shown in Supplementary Table S2. The characteristics of eligible studies were described in Table 1.

(1) A total of 453 AD animals were included, and 12 studies used transgenic mice, of which 6 studies used amyloid precursor protein (APP)/PS1 transgenic mice (He et al., 2017; Cai et al., 2018; Cai et al., 2019; Wang et al., 2021; Wu et al., 2021; Yang and Wang, 2022), 4 studies 3 × Tg AD mice (Huang et al., 2017; Chen et al., 2020; Liang et al., 2021; Ye et al., 2021), 1 study B6C3-Tg/Nju double transgenic mice (Lin et al., 2020) and 1 study TgCRND8 mice (Durairajan et al., 2012). Wistar rats were used in 5 studies (Haghani et al., 2015; de Oliveira et al., 2016; Abdel-Latif et al., 2019; Raju et al., 2021; Saleh et al., 2021), with intraperitoneal injection of scopolamine (SCO) in 2 studies (Abdel-Latif et al., 2019; Saleh et al., 2021), hippocampal injection of Aβ_1-42_ in 1 study (Haghani et al., 2015), intracerebroventricular (ICV) injection of streptozotocin (STZ) in 1 study (de Oliveira et al., 2016) and administering the heavy metal mixture in 1 study (Raju et al., 2021). Sprague Dawley (SD) rats were selected in 3 studies (Zhu and Qian, 2006; Lee et al., 2012; Hussien et al., 2018), of which administering the heavy metal mixture in 1 study (Hussien et al., 2018), intraperitoneal injection of SCO in 1 study (Lee et al., 2012) and injection peptides into the bilateral hippocampus in 1 study (Zhu and Qian, 2006). Swiss Albino mice with ICV injection of colchicine were used in 2 studies (Mangrulkar et al., 2013; Mangrulkar et al., 2016). In terms of sex, 14 studies (Zhu and Qian, 2006; Lee et al., 2012; Haghani et al., 2015; de Oliveira et al., 2016; He et al., 2017; Cai et al., 2018; Cai et al., 2019; Liang et al., 2021; Raju et al., 2021; Saleh et al., 2021; Wang et al., 2021; Wu et al., 2021; Ye et al., 2021; Yang and Wang, 2022) used male animals, 2 studies contained both sexes (Huang et al., 2017; Lin et al., 2020), only one study (Hussien et al., 2018) used female animals, and 5 studies (Durairajan et al., 2012; Mangrulkar et al., 2013; Mangrulkar et al., 2016; Abdel-Latif et al., 2019; Chen et al., 2020) did not report.
(2) For administration, 21 studies (Zhu and Qian, 2006; Durairajan et al., 2012; Lee et al., 2012; Mangrulkar et al., 2013; de Oliveira et al., 2016; Mangrulkar et al., 2016; He et al., 2017; Huang et al., 2017; Cai et al., 2018; Hussien et al., 2018; Abdel-Latif et al., 2019; Cai et al., 2019; Chen et al., 2020; Lin et al., 2020; Liang et al., 2021; Raju et al., 2021; Saleh et al., 2021; Wang et al., 2021; Wu et al., 2021; Ye et al., 2021; Yang and Wang, 2022) used oral or intragastric administration of berberine, with berberine doses ranging from 5 mg/kg/d to 260 mg/kg/d; one study (Haghani et al., 2015) utilized intraperitoneal injection of berberine at a dose of 50 mg/kg/d.
(3) In terms of behavioral outcomes, 21 studies (Zhu and Qian, 2006; Durairajan et al., 2012; Lee et al., 2012; Mangrulkar et al., 2013; Haghani et al., 2015; de Oliveira et al., 2016; Mangrulkar et al., 2016; He et al., 2017; Huang et al., 2017; Cai et al., 2018; Hussien et al., 2018; Abdel-Latif et al., 2019; Chen et al., 2020; Lin et al., 2020; Liang et al., 2021; Raju et al., 2021; Saleh et al., 2021; Wang et al., 2021; Wu et al., 2021; Ye et al., 2021; Yang and Wang, 2022) performed the Morris water maze (MWM) test. In these studies, 19 studies (Zhu and Qian, 2006; Lee et al., 2012; Mangrulkar et al., 2013; de Oliveira et al., 2016; Mangrulkar et al., 2016; He et al., 2017; Huang et al., 2017; Cai et al., 2018; Hussien et al., 2018; Abdel-Latif et al., 2019; Chen et al., 2020; Lin et al., 2020; Liang et al., 2021; Raju et al., 2021; Saleh et al., 2021; Wang et al., 2021; Wu et al., 2021; Ye et al., 2021; Yang and Wang, 2022) reported escape latency in navigation test, 14 studies (Zhu and Qian, 2006; Haghani et al., 2015; He et al., 2017; Huang et al., 2017; Cai et al., 2018; Hussien et al., 2018; Abdel-Latif et al., 2019; Chen et al., 2020; Lin et al., 2020; Liang et al., 2021; Saleh et al., 2021; Wang et al., 2021; Ye et al., 2021; Yang and Wang, 2022) reported the times of crossing platform and 14 studies (Durairajan et al., 2012; Mangrulkar et al., 2013; Haghani et al., 2015; Mangrulkar et al., 2016; He et al., 2017; Huang et al., 2017; Cai et al., 2018; Chen et al., 2020; Lin et al., 2020; Liang et al., 2021; Wang et al., 2021; Wu et al., 2021; Ye et al., 2021; Yang and Wang, 2022) reported the time spent in the target quadrant to represent the probe test.
(4) Regarding the neuropathological features of the brain in AD model animals, 10 studies (Durairajan et al., 2012; Cai et al., 2018; Hussien et al., 2018; Abdel-Latif et al., 2019; Cai et al., 2019; Lin et al., 2020; Liang et al., 2021; Saleh et al., 2021; Wu et al., 2021; Ye et al., 2021) evaluated Aβ pathological deposition, 2 studies reported (He et al., 2017; Wu et al., 2021) tau protein change, and 3 studies (de Oliveira et al., 2016; Liang et al., 2021; Ye et al., 2021) investigated the neuronal damage or apoptosis.

**TABLE 1:** Basic characteristics of the included studies.

Study (year)	Species (sex, n = treatment/model group, weight)	Modeling method	Intervention (administration, dosage, duration)	Outcomes	Intergroup differences
Zhu and Qian. (2006)	Sprague-Dawley rats (male, 6/6, 220–250 g)	Injected peptides into the bilateral hippocampuses	By Intragastric; 50 mg/kg/d; 2 weeks	1.times of crossing platform; 2.escape latency; 3.IL-1β	1.*p* < 0.05; 2.*p* < 0.05; 3.*p* < 0.05
Durairajan et al (2012)	TgCRND8 mices (N, 6/6, N)	Spontaneous AD model	By Intragastric; 100 mg/kg/d; 4 months	1.time spent in the target quadrant; 2.Aβ_1-42_; 3.GFAP	1.*p* < 0.01; 2.*p* < 0.05; 3.*p* < 0.05
Lee et al. (2012)	Sprague-Dawley rats (male, 7/7, 260–280 g)	Injected scopolamine hydrobromide into the intraperitoneal	By Intragastric; 20 mg/kg/d; 2 weeks	1.escape latency; 2.IL-1β; 3.IL-6	1.*p* < 0.01; 2.*p* < 0.05; 3.*p* > 0.01
Mangrulkar et al. (2013)	Swiss Albino mices (N, 6/6, 20–25 g)	ICV-Colchicine	By Intragastric; 40 mg/kg/d; 3 weeks	1.escape latency; 2. Time spent in the target quadrant; 3.GSH; 4.MDA	1.*p* < 0.01; 2.*p* < 0.01; 3.*p* < 0.01; 4.*p* < 0.01
Haghani et al. (2015)	Wistar rats (male, 8/8, 200–250 g)	ICV-injection of Aβ_1-42_	By Intraperitoneal; 50 mg/kg/d; 13 days	1.times of crossing platform; 2. Time spent in the target quadrant	1.*p* > 0.05; 2.*p* > 0.05
De Oliveira et al. (2016)	Wistar rats (male, 20/10, 300–350 g)	ICV-STZ	By Intragastric; 100 mg/kg/d; 3 weeks	1.escape latency; 2.Neuronal apoptosis cells; 3.AChE	1.*p* < 0.05; 2.*p* < 0.05; 3.*p* < 0.05
ShubhadaVMet al (2016)	Swiss Albino mices (N, 6/6, 20–25 g)	ICV-Colchicine	By Intragastric; 40 mg/kg/d; 3 weeks	1. Time spent in the target quadrant; 2.escape latency	1.*p* < 0.01; 2.*p* < 0.01
He et al. (2017)	APP/PS1 transgenic mices (male, 20/10, N)	Spontaneous AD model	By Intragastric; 100 mg/kg/d; 2 weeks	1. Times of crossing platform; 2.escape latency; 3.time spent in the target quadrant; 4.IL-1β; 5.GFAP; 6.MDA; 7.TNF-α; 8.Tau-ps 202; 9. Tau-ps 404	1.*p* < 0.01; 2.*p* < 0.01; 3.*p* < 0.01; 4.*p* < 0.01; 5.*p* < 0.01; 6.*p* < 0.01; 7.*p* < 0.01; 8.*p* < 0.01; 9.*p* < 0.01
Huang et al. (2017)	3× Tg AD mices (both male and female, 12/12, N)	Spontaneous AD model	By Intragastric; 100 mg/kg/d; 3 weeks	1. Times of crossing platform; 2.time spent in the target quadrant; 3.escape latency; 4.BACE1; 5.Beclin-1; 6.APP	1.*p* < 0.01; 2.*p* < 0.01; 3.*p* < 0.05; 4.*p* < 0.05; 5. *p* < 0.05; 6. *p* < 0.01
Cai et al. (2018)	APP/PS1 transgenic mice (male, 20/10, N)	Spontaneous AD model	By Intragastric; 100 mg/kg/d; 2 weeks	1. Times of crossing platform; 2.escape latency; 3. Time spent in the target quadrant; 4.Aβ_1-42_;5.BACE1; 6.APP	1.*p* < 0.05; 2.*p* < 0.05; 3.*p* < 0.05; 4.*p* < 0.01; 5.*p* > 0.05; 6.*p* < 0.01
Hussien et al. (2018)	Sprague-Dawley rats (Female, 10/10, 170–200 g)	Orally with a mixture of aluminum, cadmium, and fluoride	By Intragastric; 50 mg/kg/d; 16 weeks	1. Times of crossing platform; 2.escape latency; 3. Aβ_1-42_;4.IL-1β; 5.NO; 6.TNF-α; 7.GST; 8.GSH-Px; 9.AChE; 10.IL-6; 11.GSH	1.*p* < 0.05; 2.*p* > 0.05; 3.*p* < 0.05; 4.*p* < 0.05; 5.*p* < 0.05; 6.*p* < 0.05; 7.*p* < 0.05; 8.*p* < 0.05; 9.*p* < 0.05; 10.*p* < 0.05; 11.*p* < 0.05
Cai et al. (2019)	APP/PS1 transgenic mice (male, 6/6, 220–240 g)	Spontaneous AD model	By Intragastric; 100 mg/kg/d; 3 weeks	1. Aβ_1-42_; 2.GFAP	1.*p* < 0.05; 2.*p* < 0.05
Mohamed et al.(a)(2019)	Wistar rats (N, 8/8, 150–200 g)	Injected by 2 mg/kg BW of Scopolamine hydrobromide	By orally; 50 mg/kg/d; 4 weeks	1. Times of crossing platform; 2.escape latency; 3. Aβ_1-42_; 4.NO; 5.GST; 6.GSH-Px; 7.AChE; 8.GSH	1.*p* > 0.05; 2.*p* > 0.05; 3.*p* < 0.05; 4.*p* < 0.05; 5.*p* < 0.05; 6.*p* < 0.05; 7.*p* < 0.05; 8.*p* < 0.05
Chen et al. (2020)	3× Tg AD mices (N, 12/12, N)	Spontaneous AD model	By Intragastric; 100 mg/kg/d; 4 months	1. Times of crossing platform; 2.escape latency; 3. Time spent in the target quadrant; 4.Beclin-1	1.*p* < 0.01; 2.*p* < 0.01; 3.*p* < 0.01; 4.*p* < 0.05
Lin et al. (2020)	B6C3-Tg mice (both male and female, 11/11, N)	Spontaneous AD model	By Intragastric; 100 mg/kg/d; 12 weeks	1. Times of crossing platform; 2.escape latency; 3. Time spent in the target quadrant; 4. Aβ_1-42_; 5.BACE1; 6.IL-1β; 7.TNF-α; 8.IL-6; 9. APP	1.*p* < 0.01; 2.*p* < 0.01; 3.*p* < 0.01; 4.*p* < 0.01; 5.*p* < 0.01; 6.*p* < 0.01; 7.*p* < 0.01; 8.*p* < 0.01; 9.*p* < 0.01
Liang et al. (2021)	3× Tg AD mices (male, 24/12, N)	Spontaneous AD model	By Intragastric; 100 mg/kg/d; 16 weeks	1. Times of crossing platform; 2.escape latency; 3. Time spent in the target quadrant; 4. Aβ_1-42_; 5.BACE1; 6.APP; 7.MDA; 8. Neuronal apoptosis cells; 7.MDA; 8.APP	1.*p* < 0.01; 2.*p* < 0.01; 3.*p* < 0.01; 4.*p* < 0.01; 5.*p* < 0.01; 6.*p* < 0.01; 7.*p* < 0.01; 8.*p* < 0.01
Raju et al. (2021)	Wistar rats (male, 14/7, 180–250 g)	Administering aluminum chloride solution	By Intragastric; 100 mg/kg/d; 6 weeks	1.escape latency	1.*p* < 0.01
Saleh et al. (2021)	Wistar rats (male, 8/8, 150–200 g)	Injected scopolamine hydrobromide into the intraperitoneal	By Intragastric; 50 mg/kg/d; 4 weeks	1.escape latency; 2. Times of crossing platform; 3. Aβ_1-42_; 4.NO; 5.GST; 6.GSH-Px; 7.MDA; 8.AChE; 9.GSH	1.*p* < 0.05; 2.*p* > 0.05; 3.*p* < 0.05; 4.*p* < 0.05; 5.*p* < 0.05; 6.*p* < 0.05; 7.*p* < 0.05; 8.*p* < 0.05; 9.*p* < 0.05
Wang et al. (2021)	APP/PS1 transgenic mice (male, 10/10, N)	Spontaneous AD model	By Intragastric; 100 mg/kg/d; 3 weeks	1. Times of crossing platform; 2.escape latency; 3. Time spent in the target quadrant; 4.BACE1; 5.Beclin-1	1.*p* < 0.05; 2.*p* < 0.05; 3.*p* < 0.05; 4.*p* < 0.05; 5.*p* < 0.05
Wu et al. (2021)	APP/PS1 transgenic mice (male, 15/15, N)	Spontaneous AD model	By Intragastric; 260 mg/kg/d; 12 weeks	1. Time spent in the target quadrant; 2.escape latency; 3. Aβ_1-42_; 4.BACE1; 5.APP; 6.Tau-ps 202; 7.Tau-ps 404	1.*p* < 0.05; 2.*p* < 0.05; 3.*p* < 0.01; 4.*p* < 0.05; 5.*p* < 0.05; 6.*p* < 0.01; 7.*p* < 0.05
Ye et al. (2021)	3×Tg AD mice (male, 12/12, N)	Spontaneous AD model	By Intragastric; 100 mg/kg/d; 16 weeks	1. Times of crossing platform; 2.escape latency; 3. Time spent in the target quadrant; 4. Aβ_1-42_; 5.GFAP; 6. Neuronal apoptosis cells	1.*p* < 0.01; 2.*p* < 0.01; 3.*p* < 0.01; 4.*p* < 0.01; 5.*p* < 0.01; 6.*p* < 0.01
Yang and Wang. (2022)	APP/PS1 mice (male,10/10,N)	Spontaneous AD model	By Intragastric; 100 mg/kg/d; 16 weeks	1. Times of crossing platform; 2.escape latency; 3. Time spent in the target quadrant; 4.IL-1β; 5.TNF-α; 6.IL-6	1.*p* < 0.01; 2.*p* < 0.01; 3.*p* < 0.01; 4.*p* < 0.01; 5.*p* < 0.01; 6. *p* < 0.01

IL-1β, interleukin 1β; Aβ_1-42_, amyloid beta 42; GFAP, glial fibrillary acidic protein; IL-6, interleukin 6; GSH, glutathione; MDA, malonaldehyde; AChE, acetylcholine esterase; TNF-⍺, tumor necrosis factor ⍺; BACE1, β-site of APP, cleaving enzym; APP, amyloid precursor protein; GST, glutathione S-transferase; GPx, glutathione peroxidase; NO, nitric oxide; ICV-STZ, intracerebroventricular streptozotocin.

### 3.3 Study quality

The SYRCLE’s RoB tool was adopted to assess the quality of included studies. The scores of each study varied from 3/10 to 6/10 with an average of 3.81 points. One study (4.5%) (Lee et al., 2012) got 6 points, seventeen studies (77.2%) (Zhu and Qian, 2006; Durairajan et al., 2012; de Oliveira et al., 2016; Mangrulkar et al., 2016; He et al., 2017; Huang et al., 2017; Cai et al., 2018; Hussien et al., 2018; Abdel-Latif et al., 2019; Cai et al., 2019; Chen et al., 2020; Lin et al., 2020; Liang et al., 2021; Saleh et al., 2021; Wu et al., 2021; Yang and Wang, 2022) got 5 points, three studies (13.6%) (Mangrulkar et al., 2013; Haghani et al., 2015; Raju et al., 2021) got 4 points, one study (4.5%) (Wang et al., 2021) got 3 points. Fourteen studies (Zhu and Qian, 2006; Lee et al., 2012; de Oliveira et al., 2016; Mangrulkar et al., 2016; He et al., 2017; Cai et al., 2018; Abdel-Latif et al., 2019; Cai et al., 2019; Chen et al., 2020; Lin et al., 2020; Raju et al., 2021; Saleh et al., 2021; Wu et al., 2021; Yang and Wang, 2022) reported random allocation of animals, and the remaining eight studies (Durairajan et al., 2012; Mangrulkar et al., 2013; Haghani et al., 2015; Huang et al., 2017; Hussien et al., 2018; Liang et al., 2021; Wang et al., 2021; Ye et al., 2021) lacked information about the sequence generation process. One study (Wang et al., 2021) did not report the baseline characteristic was similar between groups. Four studies (Mangrulkar et al., 2013; Haghani et al., 2015; Raju et al., 2021; Wang et al., 2021) did not describe whether the animals were randomized during the experiments. All studies failed to report or perform blinding for caregivers or researchers. No study described the use of random outcomes for assessment. It is worth noting that the blinding for outcome assessors was mentioned in one study (Lee et al., 2012). And all studies were considered to be free of incomplete outcome data and selective outcome reporting. All studies had no other sources of bias. The complete quality assessment of included studies is shown in Supplementary Table S3.

### 3.4 Effectiveness

#### 3.4.1 Behavioral outcomes

The MWM is the most commonly used behavioral paradigm to measure cognitive ability in AD models and consists of two parts: navigation test and the spatial probe test. In the place navigation test, the escape latency (time required to find a hided platform) is recorded to evaluate the spatial learning ability of rodents. And in the probe test when the platform is absent, times of crossing platform and time spent in the target quadrant are recorded to analyze the spatial memory ability (Hernandez-Mercado and Zepeda, 2021). Of included 22 studies, 21 studies (Durairajan et al., 2012; Hooijmans et al., 2014; Jabbarzadeh Kaboli et al., 2014; Haghani et al., 2015; Ni et al., 2015; Pirillo and Catapano, 2015; de Oliveira et al., 2016; He et al., 2017; Huang et al., 2017; Wang et al., 2017; Cai et al., 2018; Hashimoto, 2018; Hussien et al., 2018; Cai et al., 2019; Fan et al., 2019; Feng et al., 2019; Wang et al., 2019; Yuan et al., 2019; Chen et al., 2020; Page et al., 2021; Zhao et al., 2021) adopted MWM.

**FIGURE 2:**
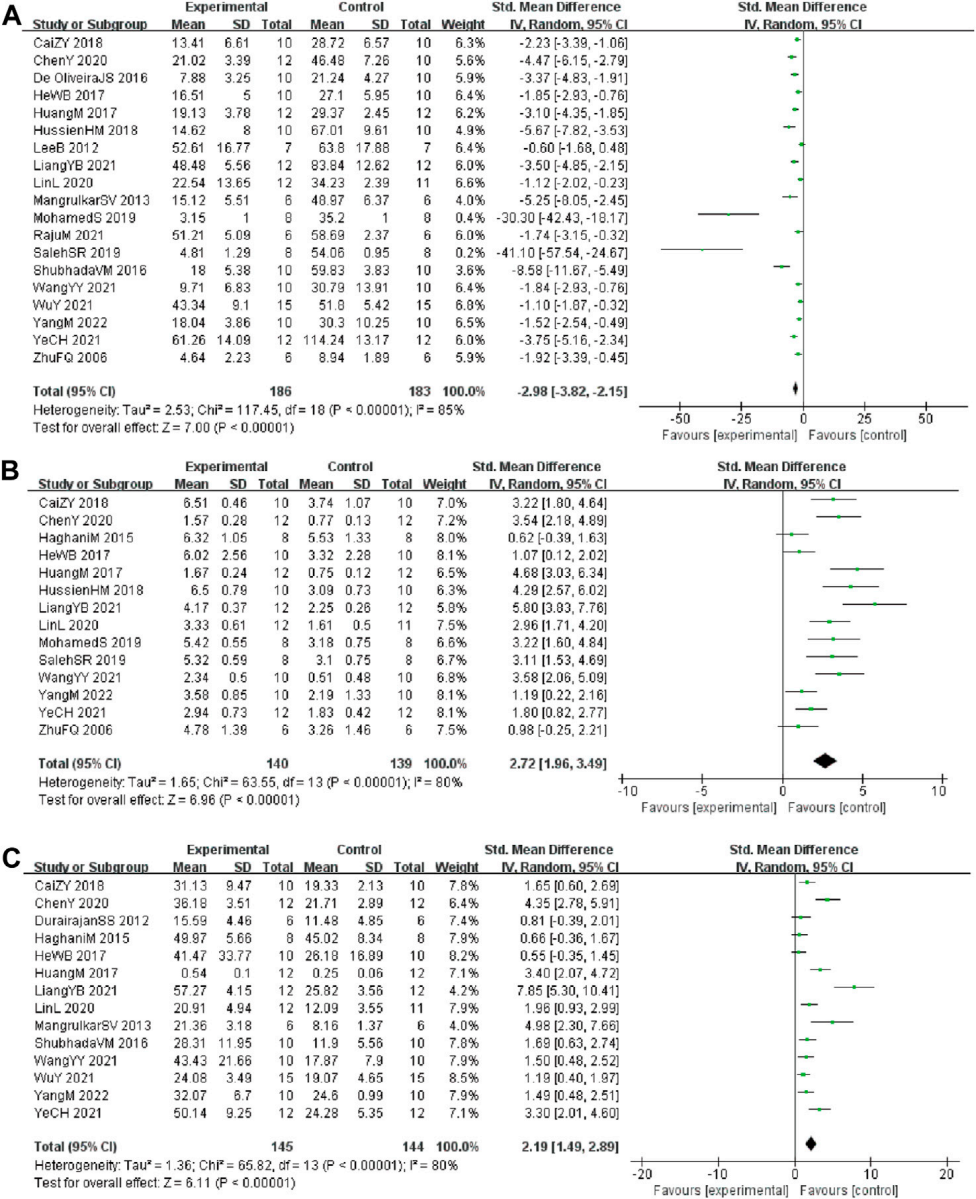
Forest plot: effect of berberine on the **(A)** escape latency, **(B)** times of crossing platform, and **(C)** time spent in the target quadrant.

Analysis of 19 studies (Durairajan et al., 2012; Hooijmans et al., 2014; Jabbarzadeh Kaboli et al., 2014; Haghani et al., 2015; de Oliveira et al., 2016; He et al., 2017; Huang et al., 2017; Wang et al., 2017; Cai et al., 2018; Hashimoto, 2018; Hussien et al., 2018; Cai et al., 2019; Feng et al., 2019; Wang et al., 2019; Yuan et al., 2019; Chen et al., 2020; Page et al., 2021; Huang et al., 2017; Hussien et al., 2018) involving 369 animals (186 in the berberine group and 183 in the control group) reported the escape latency, the analysis showed that berberine group could significantly decrease the escape latency than control group (SMD: −2.98 [95% CI: −3.82, −2.15], *p* < 0.00001, *I* ^2^ = 85%, Figure 2A)

Analysis of 14 studies (Durairajan et al., 2012; Haghani et al., 2015; Pirillo and Catapano, 2015; de Oliveira et al., 2016; Wang et al., 2017; Cai et al., 2018; Hashimoto, 2018; Hussien et al., 2018; Cai et al., 2019; Fan et al., 2019; Feng et al., 2019; Wang et al., 2019; Page et al., 2021; Zhao et al., 2021) involving 279 animals (140 in the berberine group and 139 in the control group) reported the times of crossing platform, the analysis showed that berberine group could significantly increase the times of crossing platform than control group (SMD: 2.72 [95% CI: 1.96, 3.49], *p* < 0.00001, *I* ^2^ = 80%, Figure 2B).

Analysis of 14 studies (Durairajan et al., 2012; Ni et al., 2015; Pirillo and Catapano, 2015; de Oliveira et al., 2016; He et al., 2017; Huang et al., 2017; Wang et al., 2017; Hashimoto, 2018; Cai et al., 2019; Fan et al., 2019; Feng et al., 2019; Wang et al., 2019; Chen et al., 2020; Page et al., 2021) involving 289 animals (145 in the berberine group and 144 in the control group) reported the time spent in the target quadrant, the analysis showed that berberine group could significantly increase the time spent in the target quadrant than control group (SMD: 2.19 [95% CI: 1.49, 2.89], *p* < 0.00001, *I* ^2^ = 80%, Figure 2C).

#### 3.4.2 Aβ related indicators

**FIGURE 3:**
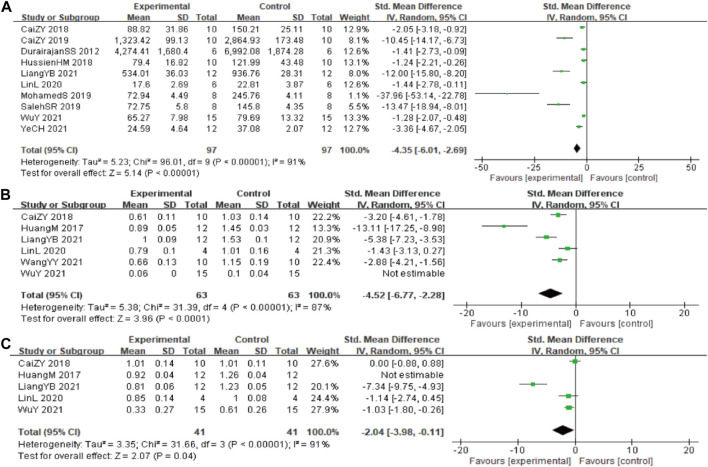
Forest plot: effect of berberine on the **(A)** Aβ_1-42_, **(B)** BACE1, and **(C)** APP.

In these studies, 10 studies (Durairajan et al., 2012; Ni et al., 2015; Yarla et al., 2016; Wang et al., 2017; Cai et al., 2018; Hashimoto, 2018; Hussien et al., 2018; Chen et al., 2020; Page et al., 2021; Zhao et al., 2021) involving 194 animals (97 in the berberine group and 97 in the control group) reported the Aβ_1-42_ quantitative, the analysis showed that berberine group could significantly decrease the Aβ_1-42_ than control group (SMD: −4.35 [95% CI: −6.01, −2.69], *p* < 0.00001, *I* ^2^ = 91%, Figure 3A).

In these studies, 6 studies (Huang et al., 2017; Cai et al., 2018; Lin et al., 2020; Liang et al., 2021; Wang et al., 2021; Wu et al., 2021) reported the β-site of APP cleaving enzyme (BACE1) levels, data from one of the studies could not be used for meta-analysis. The analysis including 5 studies (Huang et al., 2017; Cai et al., 2018; Lin et al., 2020; Liang et al., 2021; Wang et al., 2021) involving 126 animals (63 in the berberine group and 63 in the control group) showed that berberine group could significantly decrease the BACE1 than control group (SMD: −4.52 [95% CI: −6.77, −2.28], *p* < 0.0001, *I* ^2^ = 87%, Figure 3B). Five studies (Huang et al., 2017; Cai et al., 2018; Lin et al., 2020; Liang et al., 2021; Wu et al., 2021) involving 82 animals (41 in the berberine group and 41 in the control group) reported the APP levels, the analysis showed that berberine group could significantly decrease the APP than control group (SMD: −2.04 [95% CI: −3.98, −0.11], *p* = 0.04, *I* ^2^ = 91%, Figure 3C).

#### 3.4.3 Tau protein related indicators

**FIGURE 4:**
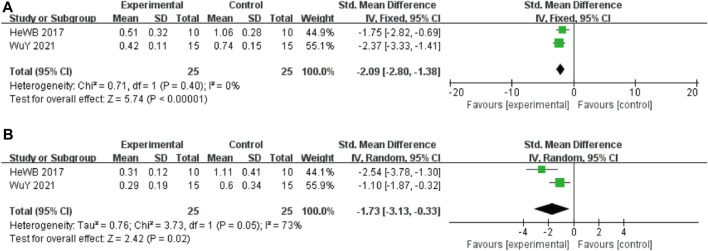
Forest plot: effect of berberine on the **(A)** Tau-ps 202 and **(B)** Tau-ps 404.

In these studies, 2 studies (He et al., 2017) involving 50 animals (25 in the berberine group and 25 in the control group) reported the Tau-ps 202 levels, the analysis showed that berberine group could significantly decrease the Tau-ps 202 levels than control group (SMD: −2.09 [95% CI: −2.80, −1.38], *p* < 0.00001, *I* ^2^ = 0%, Figure 4A). Two studies involving 50 animals (25 in the berberine group and 25 in the control group) reported the Tau-ps 404 levels, the analysis showed that berberine group could significantly decrease the Tau-ps 202 levels than control group (SMD: −1.73 [95% CI: −3.13, −0.33], *p* = 0.02, *I* ^2^ = 73%, Figure 4B).

#### 3.4.4 Oxidative stress index

**FIGURE 5:**
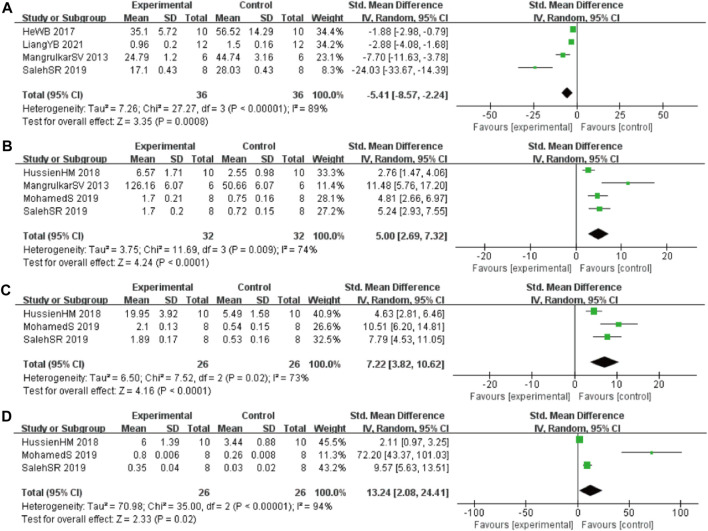
Forest plot: effect of berberine on **(A)** MDA, **(B)** GSH, **(C)** GST, and **(D)** GPx.

In these studies, 4 studies (Mangrulkar et al., 2016; He et al., 2017; Liang et al., 2021; Saleh et al., 2021) involving 72 animals (36 in the berberine group and 36 in the control group) reported the malonaldehyde (MDA) levels, the analysis showed that berberine group could significantly decrease the MDA than control group (SMD: −5.41 [95% CI: −8.57, −2.24], *p* = 0.0008, *I* ^2^ = 89%, Figure 5A). Four studies (Mangrulkar et al., 2016; Hussien et al., 2018; Abdel-Latif et al., 2019; Saleh et al., 2021) involving 64 animals (32 in the berberine group and 32 in the control group) reported the tau hyperphosphorylation (GSH) levels, the analysis showed that berberine group could significantly increase the GSH than control group (SMD: 5.00 [95% CI: 2.69, 7.32], *p* < 0.0001, *I* ^2^ = 74%, Figure 5B). Three studies (Hussien et al., 2018; Abdel-Latif et al., 2019; Saleh et al., 2021) involving 52 animals (26 in the berberine group and 26 in the control group) reported the glutathione S-transferase (GST) levels, the analysis showed that berberine group could significantly increase the GST than control group (SMD: 7.22 [95% CI: 3.82, 10.62], *p* < 0.0001, *I* ^2^ = 73%, Figure 5C). Three studies (Cai et al., 2018; Hussien et al., 2018; Zhao et al., 2021) involving 52 animals (26 in the berberine group and 26 in the control group) reported the glutathione peroxidase (GPx) levels, the analysis showed that berberine group could significantly increase the GPx than control group (SMD: 13.24 [95% CI: 2.08, 24.41], *p* = 0.02, *I* ^2^ = 94%, Figure 5D).

#### 3.4.5 Inflammatory levels

**FIGURE 6:**
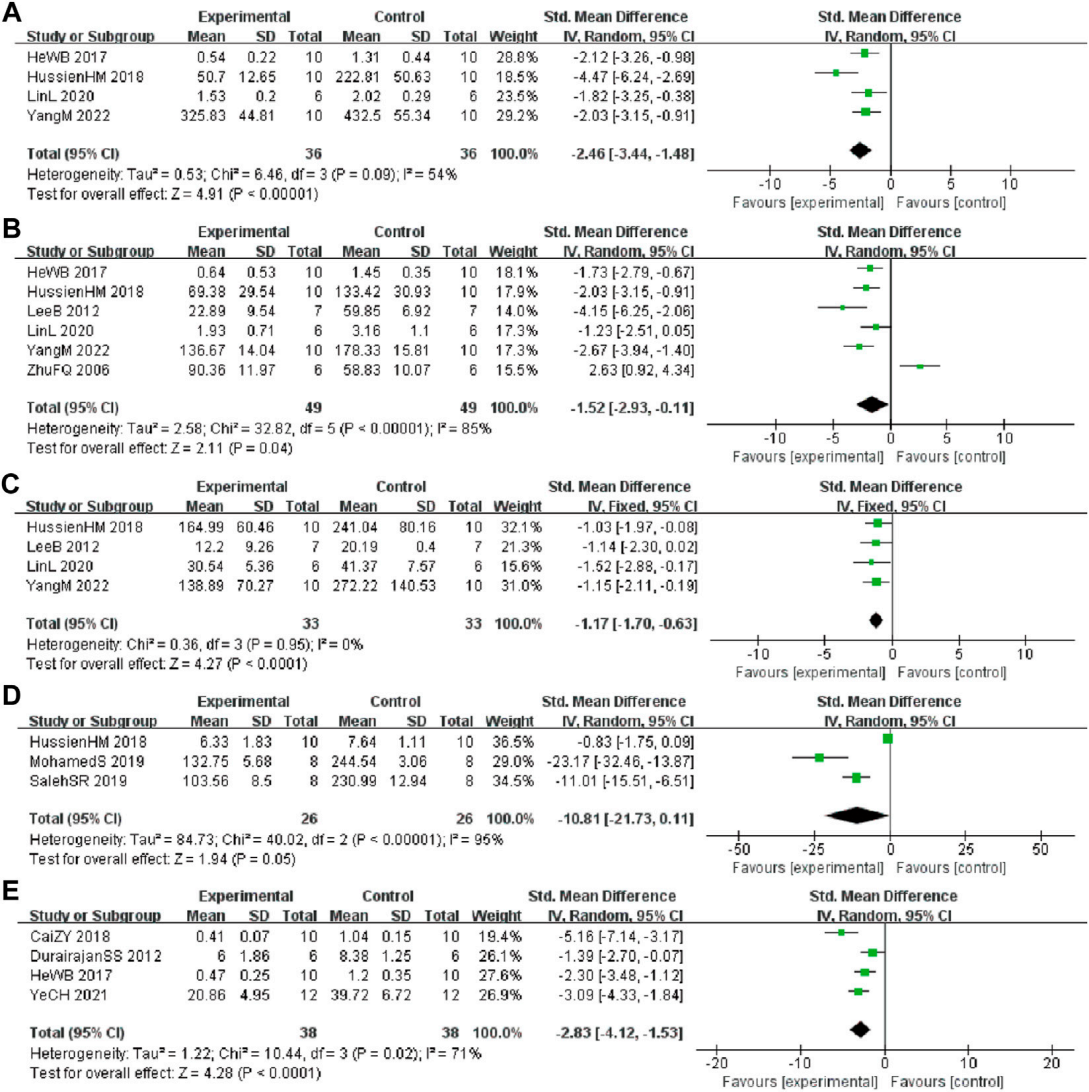
Forest plot: effect of berberine on **(A)** TNF-α, **(B)** IL-1β, **(C)** IL-6, **(D)** NO, and **(E)** GFAP.

In these studies, 4 studies (He et al., 2017; Hussien et al., 2018; Lin et al., 2020; Yang and Wang, 2022) involving 72 animals (36 in the berberine group and 36 in the control group) reported the tumor necrosis factor ⍺ (TNF-α) levels, the analysis showed that berberine group could significantly decrease the TNF-α than control group (SMD: −2.46 [95% CI: −3.44, −1.48], *p* < 0.00001, *I* ^2^ = 54%, Figure 6A). Six studies (Zhu and Qian, 2006; Lee et al., 2012; He et al., 2017; Hussien et al., 2018; Lin et al., 2020; Yang and Wang, 2022) involving 98 animals (49 in the berberine group and 49 in the control group) reported the interleukin 1β (IL-1β) levels, the analysis showed that berberine group could significantly decrease the IL-1β than control group (SMD: −1.52 [95% CI: −2.93, −0.11], *p* = 0.04, *I* ^2^ = 85%, Figure 6B). Four studies (Lee et al., 2012; Hussien et al., 2018; Lin et al., 2020; Yang and Wang, 2022) involving 66 animals (33 in the berberine group and 33 in the control group) reported the interleukin 6 (IL-6) levels, the analysis showed that berberine group could significantly decrease the IL-6 than control group (SMD: −1.17 [95% CI: −1.70, −0.63], *p* < 0.0001, *I* ^2^ = 0%, Figure 6C). Three studies (Cai et al., 2018; Hussien et al., 2018; Zhao et al., 2021) involving 52 animals (26 in the berberine group and 26 in the control group) reported the nitric oxide (NO) levels, the analysis showed that berberine group could significantly decrease the NO than control group (SMD: −10.81 [95% CI: −21.73, 0.11], *p* = 0.05, *I* ^2^ = 95%, Figure 6D). Four studies (Durairajan et al., 2012; He et al., 2017; Cai et al., 2019; Ye et al., 2021) involving 76 animals (38 in the berberine group and 38 in the control group) reported the glial fibrillary acidic protein (GFAP) levels, the analysis showed that the berberine group could significantly decrease the GFAP than control group (SMD: −2.83 [95% CI: −4.12, −1.53], *p* < 0.0001, *I* ^2^ = 71%, Figure 6E).

#### 3.4.6 Autophagy and apoptosis biomarkers

**FIGURE 7:**
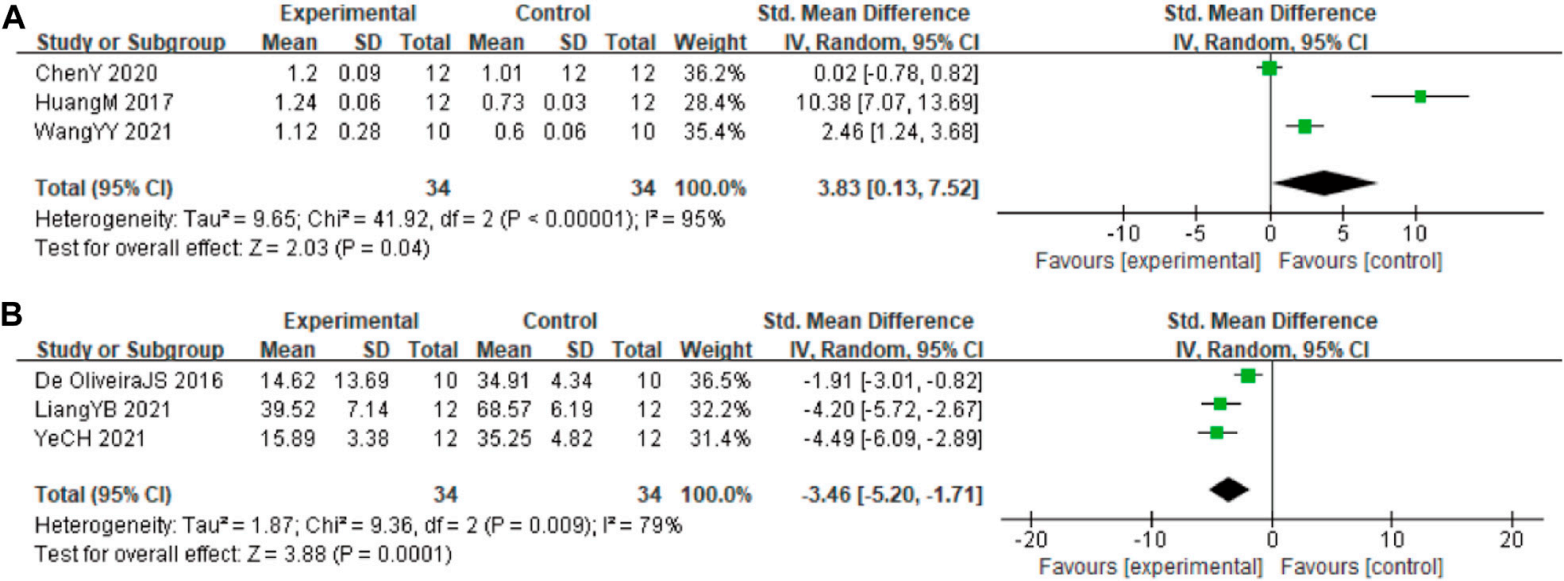
Forest plot: effect of berberine on **(A)** Beclin-1 and **(B)** neuronal apoptosis.

In these studies, 3 studies (Huang et al., 2017; Chen et al., 2020; Wang et al., 2021) involving 68 animals (34 in the berberine group and 34 in the control group) reported the Beclin-1 levels, the analysis showed that berberine group could significantly decrease the Beclin-1 than control group (SMD: 3.83 [95% CI: 0.13, 7.52], *p* = 0.04, *I* ^2^ = 95%, Figure 7A). In these studies, 3 studies (Durairajan et al., 2012; Jabbarzadeh Kaboli et al., 2014; Hashimoto, 2018) involving 68 animals (34 in the berberine group and 34 in the control group) reported neuronal apoptosis cells. The analysis showed that berberine group could significantly decrease the neuronal apoptosis cells than control group (SMD: −3.46 [95% CI: −5.20, −1.71], *p* = 0.0001, *I* ^2^ = 79%, Figure 7B).

#### 3.4.7 Cholinergic related indicators

**FIGURE 8:**
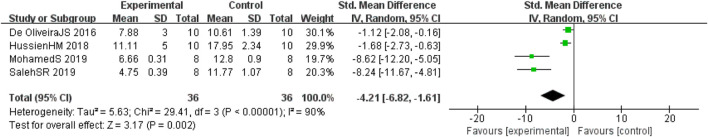
Forest plot: effect of berberine on AChE level.

Four studies (de Oliveira et al., 2016; Hussien et al., 2018; Abdel-Latif et al., 2019; Saleh et al., 2021) involving 72 animals (36 in the berberine group and 36 in the control group) reported the acetylcholine esterase (AChE) levels, the analysis showed that berberine group could significantly decrease the AChE than control group (SMD: −4.21 [95% CI: −6.82, −1.61], *p* = 0.002, *I* ^2^ = 90%, Figure 8).

### 3.5 Subgroup analysis

Because of the high heterogeneity among studies, we made the subgroup analysis of escape latency, times of crossing platform, time spent in the target quadrant and Aβ_1-42_ based on the year of publication, animal species, duration of treatment, modeling method, and dose of berberine. The results showed year of publication may be the source of heterogeneity for Aβ_1-42_. For escape latency, times of crossing platform, and time spent in the target quadrant, the subgroup analysis depending on the year of publication, animal species, modeling method, duration of treatment, and dose of berberine didn’t reveal the sources of heterogeneity among the studies. The results are presented in Supplementary Table S4.

### 3.6 Sensitivity analysis

The sensitivity analysis were conducted using a dropout-by-dropout approach, and none of the results changed direction, indicating that the overall meta-analysis was stable.

### 3.7 Publication bias

**FIGURE 9:**
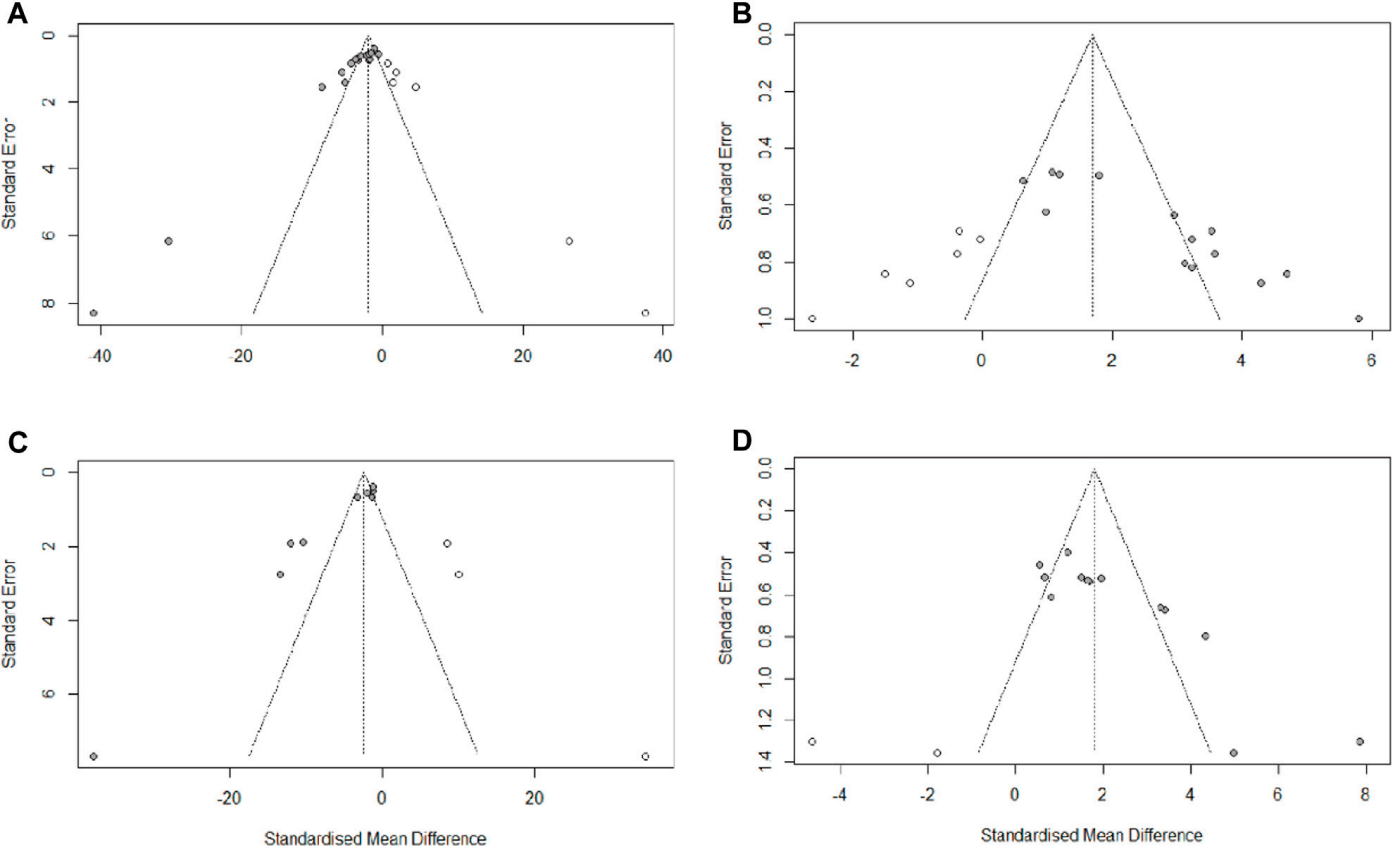
Trim-and-fill analysis for **(A)** escape latency, **(B)** times of crossing platform, **(C)** time spent in the target quadrant, **(D)** Aβ_1-42_.

We used Egger’s test to assess publication bias for escape latency, times of crossing platform, time spent in the target quadrant, and Aβ_1-42_. The results indicated that publication bias existed in all four of these observations (PEgger <0.0001). Then, asymmetry was corrected using the trim and fill method, six potential studies that may have been missed for escape latency and times of crossing platform, two potential studies that may have been missed for time spent in the target quadrant, and three potential studies that may have been missed for Aβ_1-42_. The trim and fill analysis indicated that missed studies didn’t change the magnitude of the overall pooled effect size for times of crossing platform and time spent in the target quadrant. However, the magnitude of the overall pooled effect size for escape latency and Aβ_1-42_ were altered (Figure 9). The results from Egger’s test and trim-and-fill analysis are presented in Supplementary Table S5.

### 3.8 Time-dose interval analysis

**FIGURE 10:**
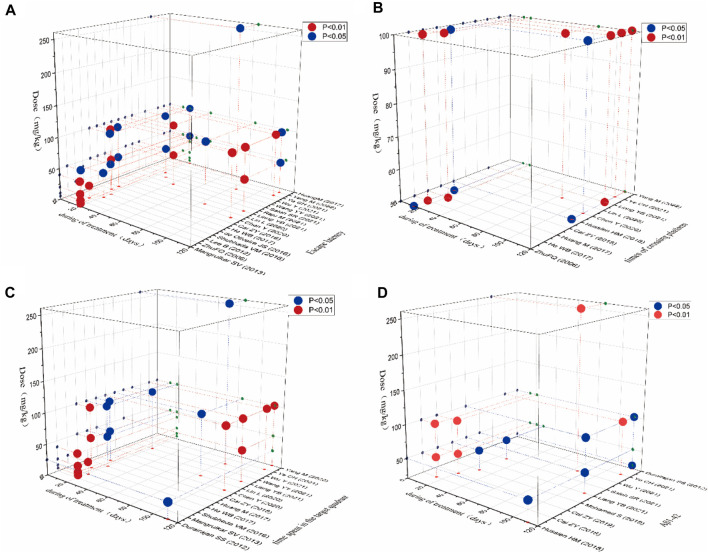
Time-dose interval analysis scatter plot for **(A)** escape latency, **(B)** times of crossing platform, **(C)** time spent in the target quadrant, **(D)** Aβ_1-42_.

In this study, “time-dose analysis” of berberine for AD was carried out for the primary indicators (Figure 10). The analysis showed that treatment with berberine at doses of 5–260 mg for 2–16 weeks had a better effect on the escape latency compared to model group (*p* < 0.05). The result showed that treatment with berberine at doses of 50–100 mg for 2–16 weeks had a better effect on the times of crossing platform compared to model group (*p* < 0.05). The analysis showed that treatment at doses of 5–260 mg with berberine for 2–16 weeks had a better effect on the time spent in the target quadrant compared to model group (*p* < 0.05). The result showed that treatment with berberine at doses of 50–260 mg for 2–16 weeks had a better effect on Aβ_1-42_ compared to model group (*p* < 0.05). Overall analysis showed that berberine had a better effect on AD when administered at doses of 5–260 mg/kg for 2–16 weeks.

## 4 Discussion

### 4.1 Effectiveness and summary of evidence

This meta-analysis evaluated the efficacy of berberine administration in preclinical models of AD. In this systematic review and meta-analysis, we included 22 studies (a total of 453 experimental animals) and performed 18 analyses (including 4 primary and 14 secondary outcome indicators). Based on the results of meta-analysis, berberine significantly shortened the escape latency, increased times of crossing platform and time spent in the target quadrant, and decreased pro-oligomerized Aβ_1-42_ deposition in animal models of AD. The above results suggested that berberine could significantly improve learning and memory ability, reduce Aβ_1-42_ accumulation, and have a therapeutic effect on reducing cognitive impairment and delaying the progression of AD. The potential mechanisms by which berberine exerts protective effects on AD animal may be closely related to anti-neuroinflammation, anti-oxidative stress, modulation of autophagy, inhibition of neuronal cell apoptosis and protection of the cholinergic system. For the primary outcome indicators with significant heterogeneity, we first combined them by random-effects models to obtain more objective results than fixed-effects models, followed by subgroup analysis to assess the effects of the variables and to explore sources of heterogeneity. Based on the results of the subgroup analysis, the type of AD model could be a source of heterogeneity in escape latency, times of crossing platform, time spent in the target quadrant, whereas year of publication could be a source of heterogeneity in Aβ_1-42_. The reduced heterogeneity observed in the subgroup analysis suggests that berberine has different effects on different AD models and publication years, and that the efficacy of berberine may vary by publication year and AD model. Notably, our subgroup analysis of drug dose, the primary outcome indicator, found that drug dose was not a source of heterogeneity. However, drug dose was measured as ≤50 mg and >50 mg as dose points for subgroup analysis, and between dose points, the effect size was larger for ≤50 mg, possibly due to the small number of studies, differences in animal species, and differences in treatment cycles.

### 4.2 Possible mechanism of berberine on AD

**FIGURE 11:**
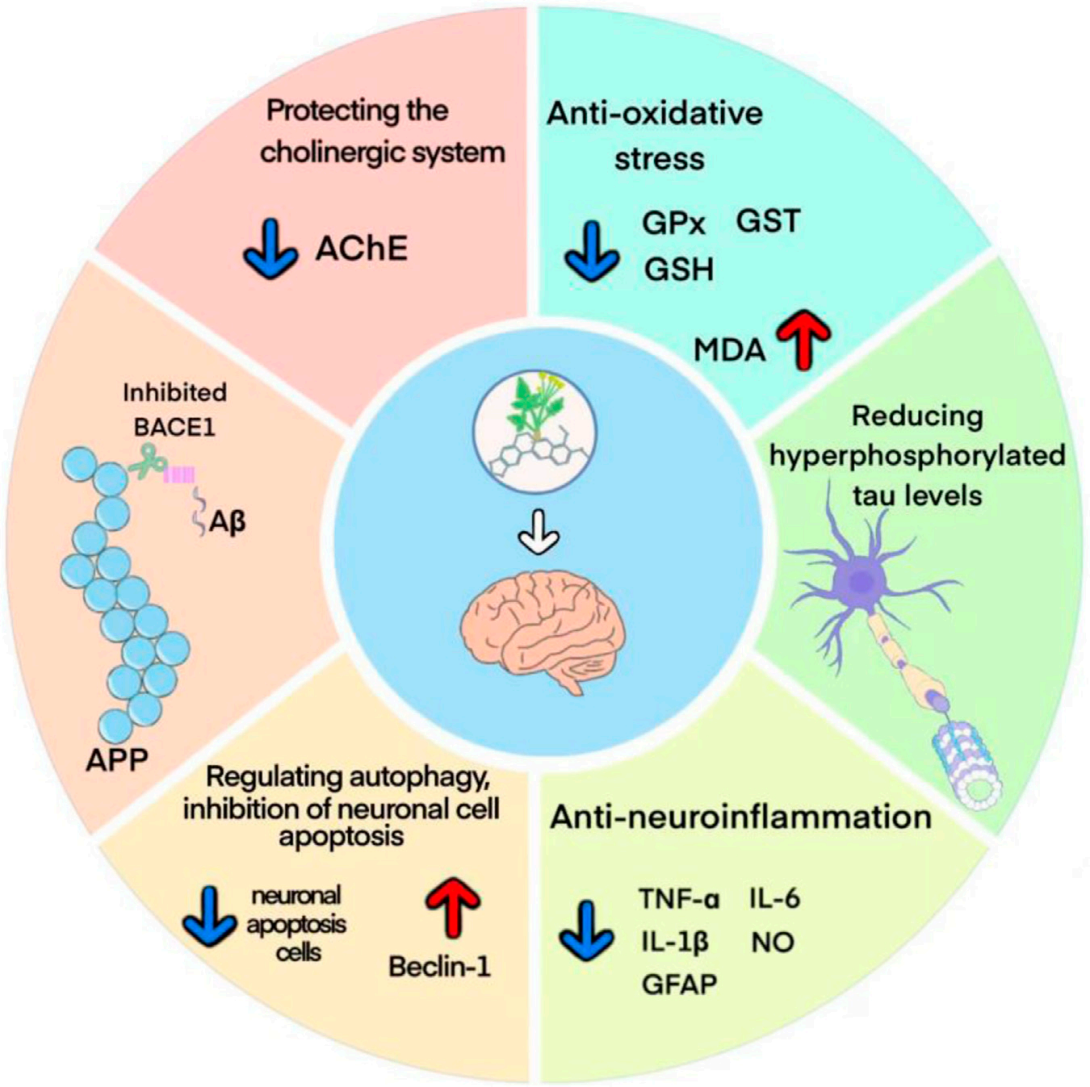
Possible mechanism of berberine on AD.

The pathogenesis of AD mainly involves Aβ deposition, tau protein hyperphosphorylation, cholinergic damage, neuroinflammation, oxidative stress and neuronal synaptic dysfunction (Yin et al., 2016). The potential protective mechanisms of berberine against AD may involve multiple molecular mechanisms, and a better understanding of these protective mechanisms may provide more theoretical basis for its clinical application (Figure 11).

The Aβ deposition is thought to be a key event in the pathogenesis of AD (Huat et al., 2019). Among them, Aβ_1-42_, which is mainly found in the brain parenchyma of AD patients, has more potent neurotoxic effects and is more likely to form plaque load than Aβ_1-40_ (Ahmed et al., 2010; Frisoni et al., 2022). Studies have shown that berberine could inhibit the Aβ_1-42_ deposition by reducing the levels of APP and BACE1 proteins. Moreover, berberine has been reported to inhibit the activity of β/γ-secretase and enhance α-secretase activity in the hippocampus of AD mice, reduce the levels of P62, Bcl-2, APP, and BACE1, and thus decrease the levels of extracellular and intracellular Aβ_1-42_ (Durairajan et al., 2012; Huang et al., 2017; Cai et al., 2018; Wu et al., 2021). NFTs are another key pathological hallmark in the brains of AD patients. Normal tau proteins are involved in microtubule assembly and stabilize synaptic transmission mechanisms (Chinthapalli, 2014). Tau protein hyperphosphorylation leads to microtubule instability and rupture, disrupting the axonal transport system, and contributing to synaptic loss and degeneration, which makes tau proteins more susceptible to aggregation and promotes NFTs formation (Otero-Garcia et al., 2022). It was shown that berberine significantly attenuated neuronal damage and reduced hyperphosphorylated tau levels at hippocampal Thr205 and Thr231 sites by regulating Akt/GSK3β pathway and phosphatase 2A protein expression (Chen et al., 2020; Wu et al., 2021; Yang and Wang, 2022).

The occurrence of AD is highly associated with neuroinflammation and oxidative stress in the brain (Seo et al., 2018). The neuroinflammatory process of AD is mainly driven by microglia and astrocytes in the brain. Early in plaque formation, Aβ produced by APP cleavage forms aggregates, and these aggregates cause phagocytosis by microglia, which releases a variety of pro-inflammatory cytokines as well as neurotoxic substances, including NO, IL-1β, IL-6, TNF-α, etc. These cytokines recruit more microglia into amyloid plaques and they are urged to release more harmful Aβ, leading to amyloid plaque growth (Liu et al., 2022). In addition, astrocytes are also capable of responding to inflammatory signals and promoting inflammation (Meda et al., 2001). GFAP, a biomarker of reactive astrocyte proliferation, has high plasma levels in preclinical AD patients and is a promising candidate biomarker for the early stages of disease (Benedet et al., 2021). The current study suggested that berberine attenuated cognitive decline and tau hyperphosphorylation by inhibiting microglia and astrocyte activation and reducing TNF-α and IL-1β expression (He et al., 2017; Hussien et al., 2018). Oxidative stress serves as a bridge connecting various mechanism of AD (Bai et al., 2022). As human life expectancy continues to increase, susceptibility to oxidative stress leads to an increase in oxidative biomarkers, and excess reactive oxygen species promotes AD progression (Ionescu-Tucker and Cotman, 2021). Studies have shown that berberine could significantly increase the activity of GSH, GPx and GST in brain tissues, while decreascing the level of MDA. It indicated that berberine could improve the antioxidant capacity of brain tissue and reduce the damage of free radicals to brain tissue, thus improving the learning and memory ability and anti-aging (He et al., 2017).

In addition, growing evidence links the regulation of autophagy to altered Alzheimer’s pathogenesis (Nafchi et al., 2022; Wang et al., 2022). The highly conserved and regulated autophagic pathway is one of the key processes in preventing and neutralizing the pathogenic accumulation of toxic proteins that may ultimately contribute to the development of neurodegenerative diseases such as AD (Diab et al., 2023). Therefore, accelerated clearance of injured or worn-out cellular constituents and proteins through increasing autophagy is expected to inhibit neuronal apoptosis and contribute to AD therapy (Islam and Tabrez, 2017). Study suggested that berberine promoted autophagic clearance of tau protein by enhancing autophagic activity, thereby reducing Aβ production and the resulting neuronal apoptosis (Chen et al., 2020). Acetylcholine (ACh), one of the major neurotransmitters in the brain (Twarowski and Herbet, 2023), plays an crucial role in maintaining learning and memory capacity (Hampel et al., 2017). In the process of aging and neuronal degeneration, the cholinergic system undergoes severe lesions, mainly manifested as the reduction of ACh synthesis, release and uptake in the brain, which is closely related to the severity of dementia (Kim et al., 2003). AChE is the specific hydrolase of ACh, and is often used as a marker to assess cholinergic damage (Gil-Bea et al., 2005). Previous studies revealed that berberine downregulated the expression of AChE and inhibits its activity in the brain through activation of liver kinase B1 (LKB1)/5′-adenosine monophosphate-activated protein kinase (AMPK) signaling, thereby attenuating Aβ pathology and rescuing synaptic damage (Hussien et al., 2018; Cai et al., 2019).

### 4.3 Limitations and considerations

Although the preclinical application of berberine for AD is promising, there are still some limitations to be considered. First, we only searched English databases of higher quality, which may lead to language bias. Second, The study data in most of the articles contained multiple dose groups, and for the sake of accuracy and authenticity of the study data, we only included data from the high dose groups, which may lead to selection bias. Third, the meta-analysis of outcome indicators was limited by high heterogeneity. Although we also attempted subgroup analysis and sensitivity analysis, still did not identify the source of heterogeneity. Fourth, the methodological quality of the included studies was generally low, with most studies referring only to randomization and not to specific randomization methods. Fifth, the effect of berberine on escape latency and Aβ_1-42_ may be overestimated due to suspected publication bias. Finally, molecular markers regulating hyperphosphorylation of Tau proteins have been less well studied.

For these reasons, we suggest that researchers should pay attention to the following elements in future animal experiments: 1) expand the sample size; 2) undertake rigorous methodological design; 3) focus on negative reports; and 4) attention to molecular markers associated with Tau proteins.

## 5 Conclusion

To sum up, our meta-analysis and systematic review suggest that berberine (5 mg/kg-260 mg/kg) could significantly improve learning and memory ability, reduce Aβ_1-42_ accumulation, and ameliorate tau protein hyperphosphorylation. The related mechanisms of action may be closely related to anti-neuroinflammation, anti-oxidative stress, modulation of autophagy, inhibition of neuronal cell apoptosis and protection of the cholinergic system. However, the results of this study should be interpreted with caution due to the overall low quality of the included preclinical studies.

## Data Availability

The original contributions presented in the study are included in the article/Supplementary Material, further inquiries can be directed to the corresponding authors.

## Abbreviations

AD, Alzheimer’s disease; Aβ, amyloid beta; NFTs, intracellular neurofibrillary tangles; TCM, Traditional Chinese Medicine; SMD, standardized mean differenc; CI, confidence interval; APP, amyloid precursor protein; SCO, scopolamine; ICV, Intracerebroventricular; STZ, streptozotocin; MWM, Morris water maze; MDA, malonaldehyde; GSH, glutathione; GST, glutathione S-transferase; GPx, glutathione peroxidase; TNF-⍺, tumor necrosis factor ⍺; IL-1β, interleukin 1β; NO, nitric oxide; GFAP, glial fibrillary acidic protein; BACE1, β-site of APP cleaving enzyme; AChE, acetylcholine esterase chemical compounds studied in this article: berberine (PubChem CID: 2353).
