# Supplementary material for: Neuroprotective effects and possible mechanisms of berberine in animal models of Alzheimer’s disease: a systematic review and meta-analysis
Source: Front Pharmacol. 2024 Jan 8;14:1287750. doi: 10.3389/fphar.2023.1287750 (PMC10800531; doi:10.3389/fphar.2023.1287750)
Supplement: Supplementary file 1 [file Table1.docx]

**Supplementary Table 1.** Literature search strategy for berberine in the treatment of AD

| No. Search Items |
| --- |
| No. Search Items |
| 1. Alzheimer Disease[Mesh] |
| 1. dementia[Title/Abstract] |
| 1. cognitive impairment[Title/Abstract] 2. β-amyloid peptide[Title/Abstract] 3. Tau protein[Title/Abstract] 4. amyloid plaques[Title/Abstract] 5. memory impairment[Title/Abstract] |
| 1. 1 OR 2 OR 3 OR 4 OR 5 OR 6 OR 7 |
| 1. Berberine[Mesh] |
| 1. Umbellatine[Title/Abstract] |
| 1. 9 OR 10 |
| 1. 8 AND 11 |
