# Supplementary material for: Neuroprotective effects and possible mechanisms of berberine in animal models of Alzheimer’s disease: a systematic review and meta-analysis
Source: Front Pharmacol. 2024 Jan 8;14:1287750. doi: 10.3389/fphar.2023.1287750 (PMC10800531; doi:10.3389/fphar.2023.1287750)
Supplement: Supplementary file 2 [file Table2.docx]

**Supplementary Table 2.** Information on berberine of each study

| **Study (years)** | **Source** | **Purity (%)** |
| --- | --- | --- |
| ZhuFQ et al (2006) | Sigama-Aldrich (St. Louis, MO, USA) | 99 |
| DurairajanSS et al (2012) | Sigama-Aldrich (St. Louis, MO, USA) | 99 |
| LeeB et al (2012) | Sigama-Aldrich (St. Louis, MO, USA) | 99 |
| MangrulkarSV et al (2013) | Sigama-Aldrich (St. Louis, MO, USA) | 99 |
| HaghaniM et al (2015) | Unknown | Unknown |
| De OliveiraJS et al (2016) | Sigma-Aldrich (St. Louis, MO, USA) | 99 |
| ShubhadaVM et al (2016) | Sigama-Aldrich (St. Louis, MO, USA) | 99 |
| HeWB et al (2017) | Hangzhou Sanofi Minsheng Health Pharmaceutical Co. Ltd., China | Unknown |
| HuangM et al (2017) | Sigama-Aldrich (St. Louis, MO, USA) | 99 |
| CaiZY et al (2018) | Hangzhou Sanofi Minsheng Health Pharmaceutical Co. Ltd., China | Unknown |
| HussienHM et al (2018) | Sigama-Aldrich (St. Louis, MO, USA) | 99 |
| CaiZY et al (2019) | Hangzhou Sanofi Minsheng Health Pharmaceutical Co. Ltd., China | Unknown |
| MohamedS et al (2019) | Sigama-Aldrich (St. Louis, MO, USA). | 99 |
| ChenY et al (2020) | Sigama-Aldrich (St. Louis, MO, USA). | 99 |
| LinL et al (2020) | Sigama-Aldrich (St. Louis, MO, USA) | 99 |
| LiangYB et al (2021) | Sigama-Aldrich (St. Louis, MO, USA). | 99 |
| RajuM et al (2021) | Sigama-Aldrich (St. Louis, MO, USA). | 99 |
| SalehSR et al (2019) | Sigama-Aldrich (St. Louis, MO, USA). | 99 |
| WangYY et al (2021) | Hangzhou Sanofi Minsheng Health Pharmaceutical Co. Ltd., China | Unknown |
| WuY et al (2021) | Unknown | Unknown |
| YeCH et al (2021) | Sigama-Aldrich (St. Louis, MO, USA). | 99 |
| YangM et al (2022) | Shanghai Ding Rui Chemical Co.,Ltd., China | 98 |
