# Supplementary material for: Neuroprotective effects and possible mechanisms of berberine in animal models of Alzheimer’s disease: a systematic review and meta-analysis
Source: Front Pharmacol. 2024 Jan 8;14:1287750. doi: 10.3389/fphar.2023.1287750 (PMC10800531; doi:10.3389/fphar.2023.1287750)
Supplement: Supplementary file 3 [file Table5.docx]

**Supplementary Table 5.** Results from Egger’s test and trim and fill analysis

| Parameter | Egger’s test  *P* value | Before trim and fill | | | After trim and fill | | |
| --- | --- | --- | --- | --- | --- | --- | --- |
|  |  | *P* value | SMD | NO. studies | *P* value | SMD | NO. studies |
| Escape latency | < 0.001 | P < 0.05 | -2.98 | 19 | P > 0.05 | -2.01 | 25 |
| times of crossing platform | < 0.001 | P < 0.05 | 2.94 | 14 | P < 0.05 | 1.69 | 20 |
| time spent in the target quadrant | < 0.05 | P < 0.05 | 2.27 | 14 | P < 0.05 | 1.80 | 16 |
| Aβ_1-42_ | < 0.001 | P < 0.05 | -3.62 | 10 | P > 0.05 | -2.47 | 13 |

NO, number; Aβ_1-42_, amyloid beta 42; SMD, standardized mean difference.
