# Supplementary material for: Neuroprotective effects and possible mechanisms of berberine in animal models of Alzheimer’s disease: a systematic review and meta-analysis
Source: Front Pharmacol. 2024 Jan 8;14:1287750. doi: 10.3389/fphar.2023.1287750 (PMC10800531; doi:10.3389/fphar.2023.1287750)
Supplement: Supplementary file 4 [file Table3.docx]

**Supplementary Table 3.** The methodological quality of included studies

| Study(year) | A | B | C | D | E | F | G | H | I | J | Total |
| --- | --- | --- | --- | --- | --- | --- | --- | --- | --- | --- | --- |
| ZhuFQ et al (2006) | ? | + | - | + | ? | ? | ? | + | + | + | 5 |
| DurairajanSS et al (2012) | - | + | - | + | ? | ? | ? | + | + | + | 5 |
| LeeB et al (2012) | ? | + | - | + | ? | ? | + | + | + | + | 6 |
| MangrulkarSV et al (2013) | ? | + | - | + | ? | ? | ? | + | + | + | 5 |
| HaghaniM et al (2015) | - | + | - | - | ? | ? | ? | + | + | + | 4 |
| De OliveiraJS et al (2016) | ? | + | - | + | ? | ? | ? | + | + | + | 5 |
| ShubhadaVM et al (2016) | - | + | - | - | ? | ? | ? | + | + | + | 4 |
| HeWB et al (2017) | ? | + | - | + | ? | ? | ? | + | + | + | 5 |
| HuangM et al (2017) | - | + | - | + | ? | ? | ? | + | + | + | 5 |
| CaiZY et al (2018) | ? | + | - | + | ? | ? | ? | + | + | + | 5 |
| HussienHM et al (2018) | - | + | - | + | ? | ? | ? | + | + | + | 5 |
| CaiZY et al (2019) | ? | + | - | + | ? | ? | ? | + | + | + | 5 |
| MohamedS et al (2019) | ? | + | - | + | ? | ? | ? | + | + | + | 5 |
| ChenY et al (2020) | ? | + | - | + | ? | ? | ? | + | + | + | 5 |
| LinL et al (2020) | ? | + | - | + | ? | ? | ? | + | + | + | 5 |
| LiangYB et al (2021) | ? | + | - | + | ? | ? | ? | + | + | + | 5 |
| RajuM et al (2021) | ? | + | - | - | ? | ? | ? | + | + | + | 4 |
| SalehSR et al (2019) | ? | + | - | + | ? | ? | ? | + | + | + | 5 |
| WangYY et al (2021) | - | ? | - | ? | ? | ? | ? | + | + | + | 3 |
| WuY et al (2021) | ? | + | - | + | ? | ? | ? | + | + | + | 5 |
| YeCH et al (2021) | - | + | - | + | ? | ? | ? | + | + | + | 5 |
| YangM et al (2022) | ? | + | - | + | ? | ? | ? | + | + | + | 5 |

A: Sequence generation; B: Baseline characteristics; C: Allocation concealment; D: Random housing; E: Blinding for trial researchers and caregivers; F: Random outcome assessment; G: Blinding for outcome assessors; H: Incomplete outcome data; I: Selective outcome reporting; J: Other sources of bias. +: low-risk of bias; ?: unclear-risk of bias; -, high-risk of bias.
