# Supplementary material for: Neuroprotective effects and possible mechanisms of berberine in animal models of Alzheimer’s disease: a systematic review and meta-analysis
Source: Front Pharmacol. 2024 Jan 8;14:1287750. doi: 10.3389/fphar.2023.1287750 (PMC10800531; doi:10.3389/fphar.2023.1287750)
Supplement: Supplementary file 5 [file Table4.docx]

**Supplementary Table 4.** The subgroup analyses of escape latency, times of crossing platform, time spent in the target quadrant, and Aβ_1-42_

| **Parameter** | **Subgroup** |  | | **No. studies** | **SMD [95% CI]** | ***I*^2^** | ***P*** |
| --- | --- | --- | --- | --- | --- | --- | --- |
| escape latency | Year | before 2018 | | 9 | -3.21 [-4.37, -2.05] | 81% | <0.00001 |
|  |  | after 2018 | | 10 | -2.81 [-4.06, -1.56] | 87% | <0.00001 |
|  | Animal species | rat | | 7 | -4.34 [-6.80, -1.87] | 91% | =0.0006 |
|  |  | mice | | 12 | -2.49 [-3.25, -1.74] | 79% | <0.00001 |
|  | Dosage | ≤ 50 mg | | 10 | -4.63 [-6.52, -2.74] | 90% | <0.00001 |
|  |  | > 50 mg | | 9 | -2.20 [-2.87, -1.52] | 85% | <0.00001 |
|  | Modeling  Method | APP/PSI transgenic | | 5 | -1.59 [-2.04,-1.15 ] | 0% | <0.00001 |
|  |  | B6C3-Tg/Nju transgenic | | 1 | -1.12 [-2.02,-0.23 ] | / | / |
|  |  | 3 × Tg transgenic | | 4 | -3.61 [-4.30,-2.91 ] | 0% | <0.00001 |
|  |  | Sco-induced | | 3 | -23.17 [-50.50,4.15 ] | 96% | =0.1 |
|  |  | STZ-induced | | 1 | -3.37 [-4.83,-1.91 ] | / | / |
|  |  | Heavy mental-induced | | 2 | -3.62 [-7.47,0.23 ] | 89% | =0.07 |
|  |  | Peptide-induced | | 1 | -1.92 [-3.39,-0.45 ] | / | / |
|  |  | Colchicine-induced | | 2 | - 6.85[-10.11,-3.58 ] | 59% | <0.0001 |
|  | Duration | ≤ 4 w | | 11 | -3.50 [-4.89, -2.10] | 87% | <0.00001 |
|  |  | > 4 w | | 8 | -2.67 [-3.70, -1.64] | 83% | <0.00001 |
| times of crossing platform | Year | | before 2018 | 6 | 2.36 [1.03, 3.69] | 85% | =0.0005 |
|  |  |  | after 2018 | 8 | 3.00 [2.09, 3.90] | 73% | <0.00001 |
|  | Animal species | | rat | 5 | 2.34 [0.93, 3.75] | 80% | =0.001 |
|  |  |  | mice | 9 | 2.94 [1.99, 3.89] | 80% | <0.0001 |
|  | Dosage | | ≤ 50 mg | 7 | 2.02 [1.12, 2.91] | 73% | <0.0001 |
|  |  |  | > 50 mg | 7 | 3.43 [2.28, 4.58] | 79% | <0.00001 |
|  | Modeling  Method | | APP/PSI transgenic | 4 | 2.15 [0.92, 3.37] | 77% | =0.0006 |
|  |  |  | 3 × Tg transgenic | 4 | 3.83 [2.08, 5.57 ] | 83% | <0.0001 |
|  |  |  | B6C3-Tg/Nju transgenic | 1 | 2.96 [1.71, 4.20 ] | / | / |
|  |  |  | Sco-induced | 2 | 3.16 [2.03,4.29 ] | 0% | <0.00001 |
|  |  |  | Aβ1-42-induced | 1 | 0.62 [-0.39, 1.63] | / | / |
|  |  |  | Heavy mental-induced | 1 | 4.29 [2.57, 6.02] | / | / |
|  |  |  | Peptide-induced | 1 | 0.98 [-0.25, 6.02] | / | / |
|  | Duration | | ≤ 4 w | 8 | 2.46 [1.42, 3.50] | 80% | <0.00001 |
|  |  |  | > 4 w | 6 | 3.10 [1.88, 4.31] | 80% | <0.00001 |
| time spent in the target quadrant | Year | | before 2018 | 7 | 1.66 [0.79, 2.53] | 73% | =0.0002 |
|  |  |  | after 2018 | 7 | 2.75 [1.62, 3.87] | 84% | <0.00001 |
|  | Animal species | | rat | 1 | 0.66 [-0.36, 1.67] | / | =0.20 |
|  |  |  | mice | 13 | 2.33 [1.59, 3.07] | 80% | <0.00001 |
|  | Dosage | | ≤ 50 mg | 5 | 2.09 [0.98, 3.20] | 75% | =0.0002 |
|  |  |  | > 50 mg | 9 | 2.27 [1.32, 3.22] | 84% | =0.00001 |
|  | Modeling  Method | | APP/PSI transgenic | 5 | 1.23 [0.81,1.64 ] | 0% | <0.00001 |
|  |  |  | 3× Tg transgenic | 4 | 4.40 [2.89,5.92 ] | 72% | <0.00001 |
|  |  |  | TgCRND8 transgenic | 1 | 0.81 [-0.39,2.01 ] | / | / |
|  |  |  | B6C3-Tg/Nju transgenic | 1 | 1.96 [0.93,2.99 ] | / | / |
|  |  |  | Aβ_1-42_-induced | 1 | 0.66 [-0.36,1.67 ] | / | / |
|  |  |  | colchicine-induced | 2 | 3.09 [-0.10,1.67 ] | 80% | 0.06 |
|  | Duration | | ≤ 4 w | 7 | 1.74 [0.92, 2.57] | 72% | <0.0001 |
|  |  |  | > 4 w | 7 | 2.68 [1.49, 3.87] | 86% | <0.0001 |
| Aβ_1-42_ | Year | | before 2018 | 3 | -1.54 [-2.19, -0.90] | 0% |  |
|  |  |  | after 2018 | 7 | -7.22 [-10.21, -4.23] | 93% |  |
|  | Animal species | | rat | 3 | -15.30 [-29.42, -1.18] | 95% | =0.03 |
|  |  |  | mice | 7 | -3.62 [-5.26, -1.98] | 89% | <0.0001 |
|  | Dosage | | ≤ 50 mg | 4 | -7.19 [-11.50, -2.89] | 93% | =0.001 |
|  |  |  | > 50 mg | 6 | -3.77 [-5.68, -1.86] | 90% | =0.0001 |
|  | Modeling  Method | | APP/PSI transgenic | 3 | -3.67[-6.35, -0.98] | 91% | =0.008 |
|  |  |  | 3 × Tg transgenic | 3 | -7.49[15.95-,0.98] | 94% | =0.08 |
|  |  |  | TgCRND8 transgenic | 1 | -1.41[-2.73, -0.09] | / | / |
|  |  |  | B6C3-Tg/Nju transgenic | 1 | -1.44 [-2.78, -0.11] | / | / |
|  |  |  | Sco-induced | 2 | -24.65[-48.56, -0.74] | 89% | 0.04 |
|  |  |  | Heavy mental-induced | 1 | -1.24[-2.21, -0.26] | / | / |
|  | Duration | | ≤ 4 w | 4 | -13.01 [-21.59, -4.42] | 94% | =0.003 |
|  |  |  | > 4 w | 6 | -2.58 [-3.98, -1.17] | 91% | =0.0003 |

NO, number; Aβ_1-42_, amyloid beta 42.
