# Supplementary material for: Neuroprotective effects and possible mechanisms of berberine in animal models of Alzheimer’s disease: a systematic review and meta-analysis
Source: Front Pharmacol. 2024 Jan 8;14:1287750. doi: 10.3389/fphar.2023.1287750 (PMC10800531; doi:10.3389/fphar.2023.1287750)
Supplement: Supplementary file 6 [file Table6.DOCX]

| **Parameter** | **Subgroup** |  | **No. studies** | **SMD [95% CI]** | ***I*^2^** |
| --- | --- | --- | --- | --- | --- |
| escape latency | Year | before 2018 | 9 | -3.21 [-4.37, -2.05] | 81% |
|  |  | after 2018 | 10 | -2.81 [-4.06, -1.56] | 87% |
|  | Animal species | rat | 7 | -4.34 [-6.80, -1.87] | 91% |
|  |  | mice | 12 | -2.49 [-3.25, -1.74] | 79% |
|  | Dosage | ≤ 50 mg | 10 | -4.63 [-6.52, -2.74] | 90% |
|  |  | > 50 mg | 9 | -2.20 [-2.87, -1.52] | 85% |
|  | Modeling  Method | non-transgenic mice | 9 | -5.10 [-7.37, -2.82] | 90% |
|  |  | transgenic mice | 10 | -2.31 [-2.98, -1.64] | 72% |
|  | Duration | ≤ 4 w | 11 | -3.50 [-4.89, -2.10] | 87% |
|  |  | > 4 w | 8 | -2.67 [-3.70, -1.64] | 83% |
| times of crossing platform | Year | before 2018 | 6 | 2.36 [1.03, 3.69] | 85% |
|  |  | after 2018 | 8 | 3.00 [2.09, 3.90] | 73% |
|  | Animal species | rat | 5 | 2.34 [0.93, 3.75] | 80% |
|  |  | mice | 9 | 2.94 [1.99, 3.89] | 80% |
|  | Dosage | ≤ 50 mg | 7 | 2.02 [1.12, 2.91] | 73% |
|  |  | > 50 mg | 7 | 3.43 [2.28, 4.58] | 79% |
|  | Modeling  Method | non-transgenic mice | 5 | 2.34 [0.93, 3.75] | 80% |
|  |  | transgenic mice | 9 | 2.94 [1.99, 3.89] | 80% |
|  | Duration | ≤ 4 w | 8 | 2.46 [1.42, 3.50] | 80% |
|  |  | > 4 w | 6 | 3.10 [1.88, 4.31] | 80% |
| time spent in the target quadrant | Year | before 2018 | 7 | 1.66 [0.79, 2.53] | 73% |
|  |  | after 2018 | 7 | 2.75 [1.62, 3.87] | 84% |
|  | Animal species | rat | 1 | 0.66 [-0.36, 1.67] | / |
|  |  | mice | 13 | 2.33 [1.59, 3.07] | 80% |
|  | Dosage | ≤ 50 mg | 5 | 2.09 [0.98, 3.20] | 75% |
|  |  | > 50 mg | 9 | 2.27 [1.32, 3.22] | 84% |
|  | Modeling  Method | non-transgenic mice | 3 | 1.99 [0.28, 3.70] | 78% |
|  |  | transgenic mice | 11 | 2.27 [1.46, 3.07] | 82% |
|  | Duration | ≤ 4 w | 7 | 1.66 [0.79, 2.53] | 73% |
|  |  | > 4 w | 7 | 2.75 [1.62, 3.87] | 84% |
| Aβ_1-42_ | Year | before 2018 | 3 | -1.54 [-2.19, -0.90] | 0% |
|  |  | after 2018 | 7 | -7.22 [-10.21, -4.23] | 93% |
|  | Animal species | rat | 3 | -15.30 [-29.42, -1.18] | 95% |
|  |  | mice | 7 | -3.62 [-5.26, -1.98] | 89% |
|  | Dosage | ≤ 50 mg | 4 | -7.19 [-11.50, -2.89] | 93% |
|  |  | > 50 mg | 6 | -3.77 [-5.68, -1.86] | 90% |
|  | Modeling  Method | non-transgenic mice | 3 | -15.30 [-29.42, -1.18] | 95% |
|  |  | transgenic mice | 7 | -3.62 [-5.26, -1.98] | 89% |
|  | Duration | ≤ 4 w | 4 | -13.01 [-21.59, -4.42] | 94% |
|  |  | > 4 w | 6 | -2.58 [-3.98, -1.17] | 91% |
